# Psychological impact of isolation due to COVID-19 among young and fit dormitory residents

**DOI:** 10.1017/S0033291720004274

**Published:** 2020-10-26

**Authors:** T. D. Gosavi, J. S. Koh, M. Rosman, K. Prasad, K. Doshi, E. S. Lim, S. E. Saffari, S. K. Goh, H. S. Ong, C. Y. Chan, D. A. De Silva, E. K. Tan

**Affiliations:** 1 Department of Neurology, National Neuroscience Institute, (Singapore General Hospital campus), Singapore, Singapore; 2 Department of Neurology, National Neuroscience Institute (Tan Tock Seng Hospital campus), Singapore, Singapore; 3 Department of Ophthalmology, Singapore National Eye Centre, Singapore, Singapore; 4 Department of Psychology, Singapore General Hospital, Singapore, Singapore; 5 Department of Integrated Care Services, Singapore, Singapore; 6 Department of Health Services and Systems Research, DUKE-NUS Medical School, Singapore, Singapore; 7 Department of Orthopaedic Surgery, Singapore General Hospital, Singapore, Singapore; 8 Department of Surgery, Singapore General Hospital, Singapore, Singapore

**Keywords:** COVID-19, Psychological impact, Dormitory residents in isolation

## Dear editor

We read with interest the recent study by Debowska and colleagues on the university students' stress, depression, anxiety, and suicidality as they faced different stages of lockdown measures during the coronavirus disease 2019 (COVID-19) pandemic in Poland (Debowska, Horeczy, Boduszek, & Dolinski, 2020). The authors found a significant increase in depression as the pandemic was progressing from more relaxed measures to the strictest restrictions. This could be explained by loss of social connectedness from greater isolation, resulting in more symptoms of depression.

We like to share our findings in a study of the psychological impact due to COVID-19 in a group of relatively young healthy men who had to isolate themselves in a dormitory in Singapore. Despite Singapore’ success in curbing the spread of COVID-19 during the early phase of the pandemic, major clusters of cases started emerging in foreign worker dormitories (custom-built residences with essential amenities to support the living and social needs of foreign workers). As of September 2020, 54 317 out of 57 516 (94.3%) total COVID-19 cases in the country were dormitory residents, and 16.8% of all the dormitory residents nationwide tested positive for COVID-19. (moh.gov.sg, situation report). There residents were mostly young and fit men who worked in construction and other service-related industries (Table 1).

**Table 1.** Baseline characteristics

Characteristic	Group	Number (139 max)	Percent
Age group	20–30	60	44.8
	30–40	56	41.8
	40–50	15	11.2
	>50	3	2.2
Gender	Male	139	100
Nationality	Bangladeshi	85	61.6
	Indian	50	36.2
	Filipino	3	2.2
Marital status	Single	50	36.2
	Married	88	63.8
Number of children	1	35	25.3
	2	27	19.5
	3	11	7.9
	4	2	1.4
	0	63	45.6
Number of people staying with you in the same household in your home country	0−2	9	6.5
	3−6	98	71
	7−10	23	16.7
	More than 10	8	5.8
Highest level of education or number of years of formal schooling	Primary	14	10.1
	Secondary	96	69.6
	A level/Diploma	14	10.1
	Degree	14	10.1
Number of years in Singapore	Less than 1	7	5.1
	1−2	10	7.2
	2−5	43	31.2
	5−10	28	20.3
	More than 10	50	36.2
Line of work	Construction	105	76.6
	Manufacturing	7	5.1
	Electronic/engineering	11	8
	Others	14	10.2

Due to the enormous risk of transmission, affected dormitories were designated as gazetted isolation areas. Movement of the resident workers in and out of the dormitories was highly restricted. Food distribution, health education and medical care were provided in every dormitory.

We selected a typical dormitory and administered the validated WHO-5 well-being index and General Anxiety Disorder-7 (GAD-7) scale to assess for depression and anxiety in 139 dormitory residents. A score of 50 or less indicated depression on WHO-5 while a cut off score of 5, 10 and 15 indicated mild, moderate and severe anxiety, respectively, on GAD-7.

For these residents, safety of family back home was the most common worry (79.6%). Other concerns such as financial security and job security were less prevalent (25.5 and 31.4%, respectively). Only 36.5% were worried about contracting the disease.

About half of the respondents felt worried (55.4%) but also hopeful (51.8%). 10.8% felt hopeless. Sadness, fear and anxiety were other emotions reported by 24.5, 28.8 and 20.1%, respectively.

When asked which form of support suited them the best, 76.8% opted for reassurance from the medical team. Talking to friends and family (54.3%), motivational videos (46.4%), reassurance by employers (42.8%) and prayers (42.8%) were other important modes which suited them.

On the WHO-5 questionnaire, based on 138 responses, 20 respondents (14.5%) screened positive for depression. Mean score was 76.3; median 82. Approximately half (54.3%) had a score above 80, indicating a healthy well-being overall. None of the demographic characteristics correlated significantly with depression or level of well-being.

On screening for anxiety using GAD-7 scale, 29 (21.0%) had mild anxiety, 20 (14.5%) had moderate anxiety and only 9 (6.5%) had severe anxiety. The remaining 57.9% had no anxiety. The mean GAD-7 score of 5 and median of 3, indicated a low level of anxiety amongst the dormitory survey respondents. Odds of severe anxiety were significantly higher amongst those who are married (*p* = 0.043) and those with children (*p* = 0.012). Other demographic characteristics and depth of COVID-19-related knowledge showed no significant association with the levels of anxiety. COVID-19 test status (positive *v.* negative and tested *v.* never tested) did not have significant relationship to well-being nor to levels of anxiety.

Psychological impact of COVID-19 was expected to be high, given the health-related, social and financial stress arising from the situation. However, the impact was surprisingly less than we expected, with 57.9% of respondents showing no anxiety objectively on GAD-7 and the overall scores of anxiety being low in the cohort (mean 5, median 3; with cut off of 5 for mild anxiety). Using WHO-5 index as a screening tool for depression, we found prevalence of depression to be 14.5%. A mean score of 76 suggested a good level of wellbeing in the cohort. Part of the lower than expected prevalence of depression could be due to the immediate availability of medical education and medical counselling at the dormitory. Nevertheless, the prevalence of anxiety and depression in our cohort was higher than that reported in a local study amongst healthcare workers in Singapore (Tan et al., 2020). In that study, 14.5% of participants screened positive for anxiety and depression was noted in 8.9%.

While there are methodological differences, our study (in a group of relatively young and healthy men who were forced to isolate when the pandemic was at its peak) findings corroborate those by Debowska et al., and suggests that increasing isolation was associated with more psychological problems. The prevalence of anxiety in our cohort was also comparable to that reported in a meta-analysis amongst COVID-19 patients (Deng et al., 2020). In our study, there were also higher odds of severe anxiety in those who were married and those with children.

While advanced age and comorbidities may be associated with greater COVID-19 mortality, the psychological impact of increasing isolation can be considerable even among relatively young and healthy individuals. Immediate access to medical support to manage these problems can reduce prevalence of anxiety and depressive symptoms. However, preventive therapies post lock down will be vital for the long-term health of those affected.

## References

[ref1] Debowska, A., Horeczy, B., Boduszek, D., & Dolinski, D. (2020). A repeated cross-sectional survey assessing university students’ stress, depression, anxiety, and suicidality in the early stages of the COVID-19 pandemic in Poland. Psychological Medicine, 1–4. doi: 10.1017/S003329172000392X PMC7556906 33004087

[ref2] Deng, J., Zhou, F., Hou, W., Silver, Z., Wong, C. Y., Chang, O., … Zuo, Q. K. (2020). The prevalence of depression, anxiety, and sleep disturbances in COVID-19 patients: A meta-analysis. Annals of the New York Academy of Sciences, 1–22. doi:10.1111/nyas.14506. PMC7675607 33009668

[ref3] Tan, B., Chew, N., Lee, G., Jing, M., Goh, Y., Yeo, L., … … Sharma, V. K. (2020). Psychological impact of the COVID-19 pandemic on health care workers in Singapore. Annals of Internal Medicine, 173(4), 317–320. doi: 10.7326/M20-1083 32251513 PMC7143149

